# Clinical and epidemiological factors associated with severe dehydration from cholera among pediatric patients in the community of Lusaka, Zambia, October 2023–May 2024

**DOI:** 10.1093/tropej/fmag022

**Published:** 2026-04-03

**Authors:** Tadatsugu Imamura, Nawa Kalima, Ilunga Chambah, Chalilwe Chungu, Khozya Zyambo, Nyuma Mbewe, Paul Msanzya Zulu, Annel Sinkala, Lalisa Nambeye, Hellen Kambole, Bob Chirwa, Roma Chilengi, Lloyd Mulenga

**Affiliations:** Japan International Cooperation Agency, Tokyo 102-8012, Japan; Center for Postgraduate Education and Training, National Center for Child Health and Development, Tokyo 157-8535, Japan; National Heart Hospital, Lusaka 10101, Zambia; Zambia Paediatric Association, Lusaka, 10101, Zambia; Zambia Paediatric Association, Lusaka, 10101, Zambia; Department of Pediatrics, University Teaching Hospital, Lusaka 10101, Zambia; Zambia Paediatric Association, Lusaka, 10101, Zambia; Department of Pediatrics, University Teaching Hospital, Lusaka 10101, Zambia; Zambia Paediatric Association, Lusaka, 10101, Zambia; Department of Pediatrics, University Teaching Hospital, Lusaka 10101, Zambia; National Public Health Institute, Lusaka 10101, Zambia; National Public Health Institute, Lusaka 10101, Zambia; Adult Infectious Diseases Center, University Teaching Hospital, Lusaka 10101, Zambia; Lusaka District Health Office, Lusaka 10101, Zambia; Lusaka District Health Office, Lusaka 10101, Zambia; University Teaching Hospital, Lusaka 10101, Zambia; National Public Health Institute, Lusaka 10101, Zambia; Adult Infectious Diseases Center, University Teaching Hospital, Lusaka 10101, Zambia

**Keywords:** Cholera, Human Immunodeficiency Virus, Severe Acute Malnutrition, Low- and Middle-income Countries, Severe dehydration, pediatric, paediatric, community, case management, risk communication and community engagement, surveillance, Lusaka, Zambia

## Abstract

Factors associated with severe dehydration from cholera among children in the community is still not fully understood. We analyzed the characteristics of the pediatric cases who were hospitalized in the community cholera treatment centers in the capital Lusaka between October 2023 and May 2024. Presence of underlying conditions (e.g. human immunodeficiency virus (HIV) infection, severe acute malnutrition), specific catchment areas (e.g. low-income residential areas), and an early phase of the outbreak was associated with increased numbers of severe cases in the community by multivariate analysis. Our study highlighted the importance of mobilization of resources and efforts aimed at enhancing surveillance, risk communication, and case management in the early phase of the outbreak for children living in high-risk areas and those with underlying conditions, in order to reduce severe cases of cholera among children in the community.

## Introduction

During the cholera outbreak in Zambia, 2023–24, the Ministry of Health set up six cholera treatment centers (CTC) in the community of the epicenter; the capital Lusaka [1]. After being assessed in those community CTCs, severe cases were referred to the tertiary care facilities [2]. Kalima *et al.* previously described the factors associated with severe outcomes among pediatric patients in the tertiary care facilities [2]. However, factors associated with severe diseases of cholera in the community were still unknown. This study aimed to determine the factors associated with severe cholera among children in the community of Lusaka.

## Methods

Retrospective data analysis was performed for pediatric patients (≤15 years old) admitted to the six community CTCs in Lusaka (Bauleni, Chawama, Chipata, George, Kanyama, and Matero), between 15 October 2023 and 31 May 2024. Case management and data collection strategies were previously described [2]. In the study, suspected cases of cholera were defined as individuals who presented more than three episodes of watery stools within 24 h [3]. Among the suspected cases, those whose stool samples tested positive for *Vibrio cholerae* by bacterial culture or polymerase chain reaction (PCR) were regarded as confirmed cases [3]. During the outbreak, CTCs in Lusaka accepted both suspected and confirmed cases as cholera patients. At admissions/visits and during hospitalizations, patient information was first recorded by health care workers in the CTCs into the paper-based forms, which were developed based on the Global Task Force on Cholera Control (GTFCC) treatment guidelines [4]. Information recorded on the paper-based forms were reviewed and digitalized by trained data officers. At admission, patients were assessed for their disease severity, and diagnosed as either “no dehydration,” “some dehydration,” or “severe dehydration” [4]. Patients with no, some, and severe dehydration were assigned with treatment plans A (oral rehydration solution (ORS) over 4 h), B (ORS over 4 h, 75 ml/kg), and C (intravenous Rinder’s lactate), respectively [3].

The digitalized data were analyzed for descriptive statistics, as well as bi-variate and multivariate analysis using logistic regression. Statistical analysis was conducted using R ver.3.5.0 (R Foundation for Statistical Computing, Vienna, Austria). The study was approved by the National Health Research Authority (NHRA-1731/22/11/2024).

## Results

**Figure 1:**
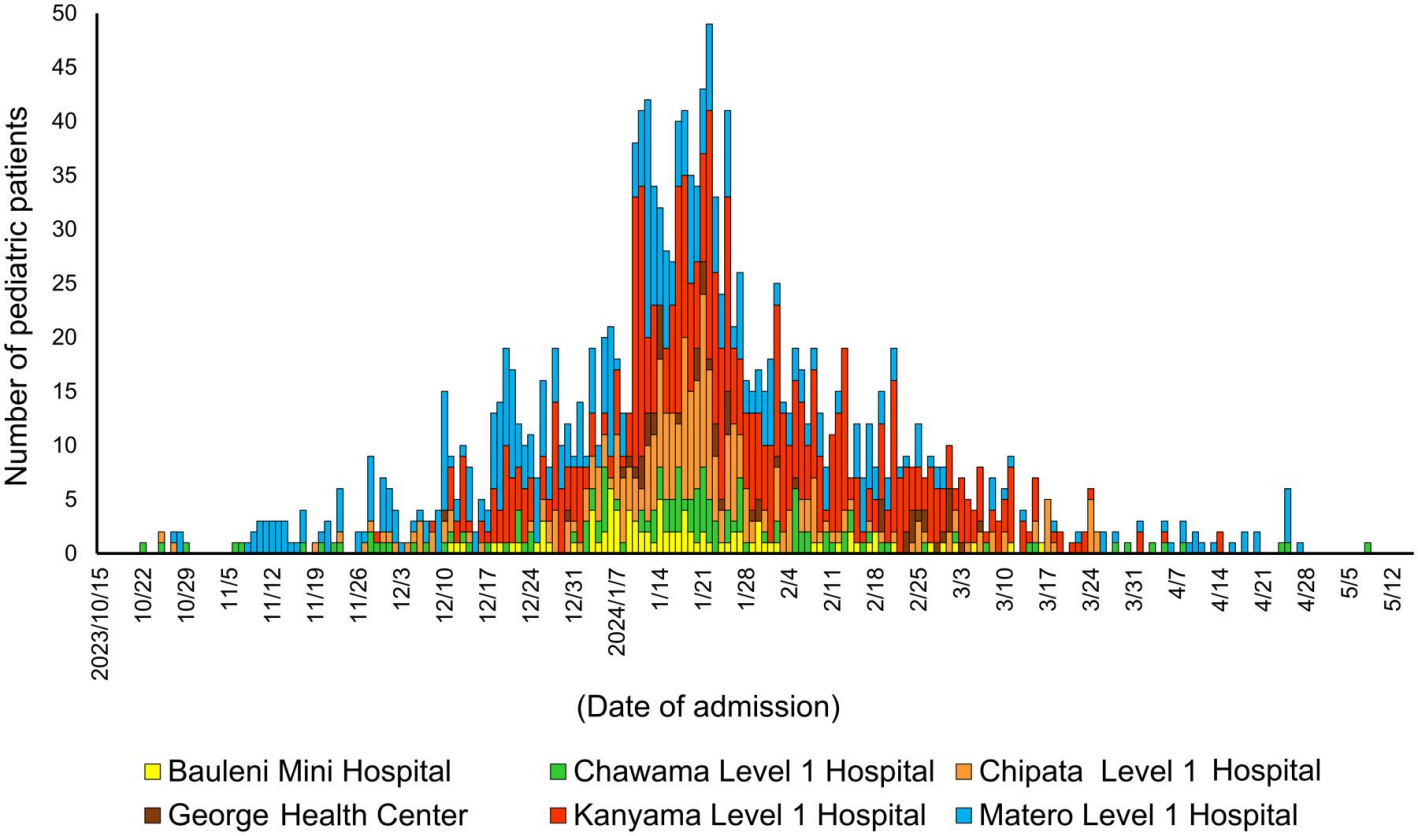
Temporal distribution of pediatric cases with cholera in the community of Lusaka, Zambia, October 2023–May 2024.

A total of 1668 patients were admitted to the community CTCs (Fig. 1). Twenty-five patients (1.6%, 25/1585) had underlying conditions, including human immunodeficiency virus (HIV) infection (*n* = 8), severe acute malnutrition (SAM) (*n* = 8), bronchial asthma (*n* = 3), sickle cell disease (*n* = 3), diabetes mellitus (*n* = 1), and tuberculosis (*n* = 1). There were 522 patients who presented with severe dehydration at admission (31.2%, 522/1640). Among these 522 cases, 290 survived to discharge, 143 were referred to the tertiary facilities, and two had fatal outcomes (Table 1).

**Table 1:** Characteristics of pediatric cholera patients and factors associated with severe dehydration in Lusaka

Characteristic	Total (*n* = 1668)	Disease severity at admission (*n* = 1640)		Factors associated with severe dehydration^a^			
				Unadjusted		Adjusted	
		Severe dehydration (*n* = 522)	No/some dehydration (*n* = 1118)	OR (95% CI)	*P*-value	OR (95% CI)	*P*-value
Age^b^ (data available for *n* = 1668)							
Age^b^ [median (IQR)]	4 (1–8)	4 (2–8)	4 (1–8)	1.01 (0.99–1.03)	0.378	1.01 (0.98–1.03)	.568
Sex (data available for *n* = 1667)							
Female	772 (46.3)	238 (45.6)	520 (46.6)	Ref	Ref	Ref	Ref
Male	895 (53.7)	284 (54.4)	597 (53.4)	1.04 (0.84–1.28)	0.717	1.01 (0.81–1.27)	.899
Underlying medical conditions (data available for *n* = 1585)							
Presence of underlying medical conditions	25 (1.6)	11 (2.2)	14 (1.3)	1.72 (0.76–3.81)	0.183	2.48 (1.03–5.83)	.038
HIV	8 (0.5)	5 (1.0)	3 (0.3)	NA	NA	NA	NA
SAM	8 (0.5)	4 (0.8)	4 (0.4)	NA	NA	NA	NA
Vaccination history (data available for *n* = 700)							
Oral cholera vaccine prior to admission	111 (15.9)	29 (13.6)	81 (16.9)	NA	NA	NA	NA
History of illness^b^ (data available for *n* = 1430)							
Number of days with watery stool	1.0 (1.0–2.0)	1.0 (1.0–2.0)	1.0 (1.0–2.0)	NA	NA	NA	NA
Number of days with vomiting	1.0 (1.0–2.0)	1.0 (1.0–2.0)	1.0 (1.0–2.0)	NA	NA	NA	NA
Cholera treatment centers (data available for *n* = 1668)							
Bauleni	92 (5.5)	25 (4.8)	66 (5.9)	Ref	Ref	Ref	Ref
Chawama	134 (8.0)	66 (12.6)	63 (5.6)	2.77 (1.57–4.98)	<0.001	2.60 (1.39–4.98)	0.003
Chipata	282 (16.9)	113 (21.6)	164 (14.7)	1.82 (1.09–3.10)	0.024	1.79 (1.03–3.19)	0.044
George	58 (3.5)	9 (1.7)	46 (4.1)	0.52 (0.21–1.17)	0.128	0.51 (0.20–1.21)	0.135
Kanyama	635 (38.1)	230 (44.1)	399 (35.7)	1.52 (0.95–2.52)	0.092	1.43 (0.85–2.50)	0.187
Matero	467 (28.0)	79 (15.1)	380 (34.0)	0.55 (0.33–0.94)	0.024	0.44 (0.25–0.79)	0.005
Year of admission (data available for *n* = 1662)							
2023 (October–December)	333 (20.0)	118 (22.7)	207 (18.6)	1.29 (1.00–1.66)	0.050	1.86 (1.40–2.47)	<0.001
2024 (January–May)	1329 (79.8)	401 77.3)	908 (81.4)	Ref	Ref	Ref	Ref
Disease severity at admission (data available for *n* = 1640)							
No dehydration	462 (28.2)	(–)	(–)	NA	NA	NA	NA
Some dehydration	656 (40.0)	(–)	(–)	NA	NA	NA	NA
Severe dehydration	522 (31.2)	(–)	(–)	NA	NA	NA	NA
Initial treatment plan at admission^c^ (data available for *n* = 1657)							
A	478 (28.8)	5 (1.0)	463 (41.5)	NA	NA	NA	NA
B	637 (38.4)	3 (0.6)	629 (56.4)	NA	NA	NA	NA
C	542 (32.7)	513 (98.5)	24 (2.2)	NA	NA	NA	NA
Outcomes (data available for *n* = 1430)							
Discharged	1100 (76.9)	290 (66.7)	792 (81.4)	NA	NA	NA	NA
Fatal	4 (0.3)	2 (0.5)	1 (0.1)	NA	NA	NA	NA
Referred	326 (22.8)	143 (32.9)	180 (18.5)	NA	NA	NA	NA

Ref, reference; OR, odds ratio; CI, confidence interval; NA, not applied; HIV, human immunodeficiency virus; SAM, severe acute malnutrition.

a The OR (95% CI) of pediatric patients with the characteristics are shown. The OR were calculated by binary and logistic regression analysis. A *P*-value less than .05 was considered as statistically significant.

b Median (interquartile range; IQR) was indicated for the age and the number of days with watery stool. Number (%) was indicated for other variables.

c Patients with no, some, and severe dehydration were assigned with treatment plans A (oral rehydration solution (ORS) over 4 h), B (ORS over 4 h, 75 ml/kg), and C (intravenous Rinder’s lactate), respectively.

In the multivariate analysis, underlying conditions (odds ratio; OR 2.48, 95% confidence interval; CI 1.03–5.83), the catchment areas being Chawama (OR 2.60, 95% CI 1.39–4.98), or Chipata (OR 1.79, 95% CI 1.03–3.19), and the year of admission being 2023 (OR 1.86, 95% CI 1.40–2.47) showed positive correlations (Table 1).

## Discussion

Our results showed that severe dehydration was more prevalent in the early phase of the outbreak (late 2023) than after the new year (early 2024). This greater proportion of cases with severe dehydration in 2023 than 2024 might have been because the public health interventions were still in the preparatory stage in 2023. Such interventions (e.g. the risk communication and community engagement activities in the outbreak areas) were intensified as the number of cholera cases surged in Lusaka after December 2023 [2, 5]. Factors including inadequate health-related knowledge and less exposure to health communications may have been contributory, especially in the early phase of the outbreak, although this was not specifically investigated in this study [6].

In addition, pediatric cases with severe dehydration at admission were linked to specific catchment areas (i.e. Chawama, Chipata). Residents in these areas might have had preexisting risk factors for developing severe diseases. All community CTCs were strategically established in the high-risk areas for cholera morbidity and mortality (e.g. low-income residential areas). However, Chipata and Chawama were particularly unique, as in the past they were reported as areas with fewer residents with education higher than primary level and adequate access to water and sanitation facilities [7]. In the report, Hamukale *et al.* documented that such spatial factors might have underlaid the geographical distribution of the community deaths associated with the coronavirus disease 2019 (COVID-19). This data might suggest that residents in those areas had high-risks for developing severe diseases in the pre-hospital/community settings during epidemics of various infectious diseases, including cholera [7]. Underlying conditions of residents in such areas (e.g. HIV, SAM) might also be assumed to have contributed to the increased number of severe cases [8].

### Strengths and limitations

The strength of the study was the use of the real-world data from the largest cholera outbreak in Zambia’s history, and a large number of cases in the dataset and was limited by an incomplete dataset, and a lack of information on socioeconomic status and cholera vaccination status.

## Conclusion

Severe cholera among children presenting to community CTCs was a feature of the early part of the 2023–24 Zambia Cholera outbreak and associated with high-risk areas with low social economic status and having an underlying condition. Our results highlight the importance of efforts to reduce severe cases of cholera among children by mobilization of resources and efforts aimed at enhancing surveillance, risk communication, and case management in the early phase of the outbreak for children living in high-risk areas and those with underlying conditions.

## Data Availability

Dataset used for this study will be shared by the study group with a reasonable request.
